# Resolutions by the Galveston Club. Resolutions by the Travis County Medical Society, and Also by the West Texas Medical Society

**Published:** 1890-11

**Authors:** 


**The Galveston Medical Club** recently adopted the following resolutions:

"*Resolved*, That the Galveston Medical Club commend R. M. Swearingen, M. D., as an able and advanced sanitarian, and endorse his six years administration of the office of State Health Officer of Texas."

"*Resolved*, That a committee of four be appointed to memorialize the Governor to appoint him State Health Officer upon the expiration of the term of the present incumbent."

The committee appointed in pursuance of the above consists of Drs. J. F. Y. Paine, B. E. Hadra, C. W. Truheart and H. A. West.

The West Texas District Medical Association met in San Antonio on the 6th instant, and passed the following resolution:

"By unanimous vote of the West Texas Medical Association it was resolved, that Dr. R. M. Swearingen, of Austin, be, and he is hereby requested to allow his name to be presented to the incoming Governor of the State for the appointment of State Health Officer, knowing that no more efficient officer, no man of purer integrity, and no more competent professional man can be found to fill that office, which he has already filled with advantage to the people and honor to himself."

[He was State Health Officer three terms, of two years each, and served under two administrations.—Ed.]

The Travis County Medical Society met in Austin on the 6th inst., and adopted similar resolutions as those of the Galveston Medical Club and the West Texas Medical Society, and appointed Drs. F. E. Daniel, T. J. Bennett and M. A. Taylor a committee to co-operate with the Galveston committee to memorilize the Governor to make the appointment.

We learn from a prominent physician from North Texas who has recently been in the principal cities in North Texas and conversed with the leading physicians there, that there is a very general—almost unanimous sentiment in favor of Dr. Swearingen's appointment.

The JOURNAL is authorized to state that any report to the effect that he will not be an applicant for the office is unfounded. Such report is circulated perhaps for an unworthy purpose, and should not be credited. Drs. Cunningham and Harrison are also in the race, and both good men.

